# DNA methylation is enhanced during Cd hyperaccumulation in *Noccaea caerulescens* ecotype Ganges

**DOI:** 10.1007/s11356-022-23983-w

**Published:** 2022-11-10

**Authors:** Serena Galati, Giovanni DalCorso, Antonella Furini, Rosaria Fragni, Chiara Maccari, Paola Mozzoni, Gianluigi Giannelli, Annamaria Buschini, Giovanna Visioli

**Affiliations:** 1 Department of Chemistry, Life Sciences and Environmental Sustainability, University of Parma, Parma, Italy; 2 Department of Biotechnology, University of Verona, Verona, Italy; 3 SSICA, Experimental Station for the Food Preserving Industry, Parma, Italy; 4 Department of Medicine and Surgery, University of Parma, Parma, Italy; 5 Centre for Research in Toxicology (CERT), University of Parma, Parma, Italy

**Keywords:** Hyperaccumulators, *Noccaea caerulescens*, Cadmium toxicity, Comet assay, Methy-sens comet assay, Antioxidant activity, Epigenetic regulation

## Abstract

In this study, we assess the DNA damage occurring in response to cadmium (Cd) in the Cd hyperaccumulator *Noccaea caerulescens* Ganges (GA) vs the non-accumulator and close-relative species *Arabidopsis thaliana*. At this purpose, the alkaline comet assay was utilized to evaluate the Cd-induced variations in nucleoids and the methy-sens comet assay, and semiquantitative real-time (qRT)-PCR were also performed to associate nucleus variations to possible DNA modifications. Cadmium induced high DNA damages in nuclei of *A. thaliana* while only a small increase in DNA migration was observed in *N. caerulescens* GA. In addition, in *N. caerulescens* GA, CpG DNA methylation increase upon Cd when compared to control condition, along with an increase in the expression of *MET1* gene, coding for the DNA-methyltransferase. *N. caerulescens* GA does not show any oxidative stress under Cd treatment, while *A. thaliana* Cd-treated plants showed an upregulation of transcripts of the respiratory burst oxidase, accumulation of reactive oxygen species, and enhanced superoxide dismutase activity. These data suggest that epigenetic modifications occur in the *N. caerulescens* GA exposed to Cd to preserve genome integrity, contributing to Cd tolerance.

**Supplementary Information:**

The online version contains supplementary material available at 10.1007/s11356-022-23983-w.

## Introduction

The heavy metal cadmium (Cd) is a widespread element, deriving from both natural sources and anthropogenic activities. The primary natural source of Cd is the phenomenon of geological weathering, while the anthropogenic sources include disposal of urban refuse, mining and metal manufacturing, smelting, and application of synthetic phosphate fertilizers (Haider et al. 2021). In 1993, the International Agency for Research on Cancer (IARC) has designated Cd as a carcinogen of Class I, and it is one of the most dangerous heavy metal pollutants. It has no physiological function both in animals and plants, except for the alga *Thalassiosira weissflogii*, in which Cd can substitute Zn in the active center of the carbonic anhydrase CDCA1 maintaining the enzymatic activity (Lane et al. 2005).

Being a non-redox active metal, Cd seems not directly cause free radicals by the Haber–Weiss cycle or the Fenton oxidation–reduction reactions; however, the indirect formation of a variety of reactive oxygen species (ROS) such as hydrogen peroxide, hydroxyl radical, and superoxide has been reported (Cuypers et al. 2010). ROS production may interfere with the cellular antioxidant defense mechanisms, causing a change in the intracellular redox status. When it occurs, the harmful effects of Cd show as an oxidative stress (Jomova and Valko 2011). A variety of biological processes are inhibited by the Cd-induced oxidative stress, such as the mitochondrial and chloroplast activities, and this inhibition is usually ascribed to the Cd displacement of redox-active metals such as Mg or Mn, the depletion of antioxidants and the inactivation of antioxidant enzymes (Haider et al. 2021). Cd stress also perturbs both endocytosis and exocytosis disrupting actin filaments responsible for vesicle transport (De Caroli et al. 2020). In addition, Cd-induced ROS frequently impacts on the chemical or physical structures of DNA and induce both genotoxic and cytotoxic stresses, thus reducing genome stability.

As sessile organisms, plants have evolved protective mechanisms to cope with high Cd concentrations in soils, such as the prevention of Cd entry in the roots, the immobilization of Cd in vacuoles, and the production of Cd-complexing compounds, such as phytochelatins (Guan et al. 2018; Huang et al. 2020; Sebastian and Prasad 2018). These processes are dependent on the expression of specific genes and thus are controlled by genetic programming. Apart from transcriptional responses, epigenetic modifications are also essential for maintenance of plant genome stability under genotoxic metal stress (Dutta et al. 2018; Niekerk et al. 2021).

DNA methylation is one of the most common epigenetic traits and it is involved in processes such as the induction of phenotypic alterations and maintenance of genome stability when plants are under abiotic stress (Liu and He 2020). To date, only a few studies have focused on the relationship between Cd and the modulation of DNA methylation in plants (Fan et al. 2020; Feng et al. 2016; Wang et al. 2016; Shafiq et al. 2019). For instance, leaves of *Phytolacca americana* seedling showed a decrease in SOD activity upon Cd treatment, and a contemporary amplified oxidative damage, evaluated in terms of increased amount of H_2_O_2_, malondialdehyde (MDA) and 8-hydroxy 2 deoxyguanosine (8-OHdG) (Jing et al. 2022). The same authors identified a link between Cd sensing, ROS production, and the expression of DNA methylation-related genes. In more details, the expression of three chromomethylase genes was upregulated upon Cd stress, and such upregulation was abolished by the pretreatment with ROS inhibitors, pointing to ROS as mediator between Cd sensing and DNA methylation (Jing et al. 2022). The heavy metal stress is therefore able to modulate DNA methylation or demethylation, by enhancing ROS production and activity, the latter greatly depending on the metal considered, its concentration, and the plant species (Jing et al. 2022). Literature on the topic reports conflicting results, and all cases are represented, with Cd enhancing, reducing, or non-influencing the DNA methylation level in a way highly dependent on the species considered and the methylation context investigated (Singh et al. 2021), pointing out that DNA methylation modulated by heavy metals is a particularly complicated phenomenon and not a priori predictable.

Metal hyperaccumulators are represented by more than 600 plant species widespread in the world; they share the ability to concentrate metals and metalloids in their tissues without undergoing signs of toxicity (www.hyperaccumulators.org and Reeves et al. 2018). Most known taxa accumulate Ni but few taxa are capable of accumulating Cd 100 times more than the level of toxicity of non-tolerant plants (Manara et al. 2020). To better understand the role of DNA methylation in metal tolerance and toxicity, metal hypertolerant and hyperaccumulating species can be used as models (Galati et al. 2021; Gullì et al. 2018). In particular, the cruciferous plant *Noccaea caerulescens* is a well-known metal hyperaccumulator. In nature, there are populations with pronounced differences and well-defined phenotypes in tolerance and accumulation of different metals such as Zn, Cd, and Ni. In particular, the Ganges (GA) ecotype has shown superior Cd accumulation and hyper-tolerance when compared to other populations studied so far (Assuncão et al. 2003). In a previous work, we used alkaline and methy-sens comet assays to show that the Cd hyperacculator and hypertolerant species *Arabidopsis halleri,* differently to the non-tolerant species *Arabidopsis thaliana*, condenses its nucleus by DNA methylation in response to Cd treatment, suggesting a role of DNA methylation for proper plant development (Galati et al. 2021). In this work, we compared the Cd response in *N. caerulescens* GA vs the non-tolerant *A. thaliana* analyzing i) the possible DNA damages by alkaline comet assay; ii) the modulation of DNA methylation by methy-sens comet assay; iii) the superoxide dismutase (SOD) activity and ROS production, measuring the three oxidative stress biomarkers, H_2_O_2_, 7,8-Dihydro-8-oxo-2′-deoxyguanosine (8-oxo-dG) and superoxide anion; and iv) the expression of genes involved in symmetric and non-symmetric DNA methylation and genes coding for enzymes linked to oxidative stress response. The data obtained evidenced a stress response upon Cd treatment in *A. thaliana* plants with an upregulation of transcripts of the respiratory burst oxidase, accumulation of ROS, and enhanced SOD activity while no signs of oxidative stress were evidenced in *N. caerulescens* GA; meanwhile, we confirmed our previous data on the hyperaccumulator species *A. halleri* underlining the possible role of DNA methylation in shaping the Cd hyperaccumulator phenotype.

## Material and methods

### Plant material and growth conditions

Seeds of *Noccaea caerulescens* GA, which is superior to other populations to translocate Cd efficiently from the root to the shoot, (kindly provided by Prof. Aarts) and seeds of *Arabidopsis thaliana* (ecotype Columbia) were surface sterilized in 50% (v/v) sodium hypochlorite for 10 min and rinsed few times in sterile water. The sterile seeds were distributed on agarized plates containing 1 × MS medium (Murashige and Skoog 1962) and stored in the dark at 4 °C for 20 days for *N. caerulescens* and 3 days for *A. thaliana* to synchronize germination. Plantlets were grown in growth chamber under controlled conditions (22 °C; 16-h/8-h light/dark; 120 mol m^−2^ s^−1^ light and 75% HR). When the root apparatus was fully developed, plants were transferred into 3 L PE pots containing 1 × standard Hoagland’s solution and placed in a growth chamber under controlled conditions (19 °C; 16-h/8-h light/dark; 120 µmol m^−2^ s^−1^ light and 75% RH). The nutrient solution was kept aerated by air pumping and replaced weekly. After 1 week, six plants of *N. caerulescens* GA were treated with 50 μM CdSO_4_ and six plants of *A. thaliana* were treated with 5 μM CdSO_4_ for 1 week. Control plants were grown in 1 × Hoagland solution without addition of Cd.

At the end of the experiments, three leaves similar in size, from treated and untreated plants both *N. caerulescens GA* and *A. thaliana*, were collected and subjected to the alkaline comet assay, the methy-sens comet assay, and the SOD activity test. Other leaves were frozen in liquid nitrogen and stored at –80 °C for RNA extractions, or oven-dried at 70 °C for 3 days for metal quantification. Leaf samples were also collected for the in-gel SOD activity test and to determine in vivo H_2_O_2_ and O_2_˙^–^ production and processed as described in the following paragraphs to extract genomic DNA to evaluate the amount of 8-oxo-dG.

### Chemical analyses

About 0.1 g of oven-dried samples, weighted directly into quartz vessels, and rehydrated with 800 µL of high-purity deionized water (0.05 μScm^−1^, Purelab® Ultra ELGA, High Wycombe, UK) were mineralized in duplicate with 2 mL of the solution HNO_3_:HCl:H_2_O, 3:1:1 v v^−1^v^−1^ by UltraWAVE (UltraWAVE Milestone, Sorisole, Italy). HNO_3_ (67–69% mv^−1^, Chem-Lab NV, Pico-Pure Plus, Zedelgem, Belgium) and HCl (32–35% m v^−1^, VWR, NORMATOM®, Leuven, Belgium) were ultrapure grade. The operating conditions used for the mineralization are previously described (Gullì et al.^ 2018^).

At the end of the digestion, the extracts were transferred to graduated PP tubes (DigiTUBEs, SCP Science, Champlain, NY, USA) adjusting the volume to 10 mL with high-purity deionized water, filtered on 0.45-μm filters (Millex®-HA, Millipore, Merck, Darmstadt, Germany) and finally subjected to Inductively Coupled Plasma (ICP) analysis for Cd, Fe, Mn, and Zn quantification. Ten/20-fold further dilutions were necessary for some samples with higher content of Mn and Zn. The analytic measurements were carried out by an ICP-OES spectrometer (Vista-MPX, Varian, Agilent Technologies, Santa Clara, CA); the instrument configuration and measurement conditions were previously described (Gullì et al. 2018).

A mono-element solution containing 1000 mg L^−1^ of Cd (TraceCERT® Fluka Analytical, Sigma-Aldrich, St. Louis, Missouri, USA) and a multi-element solution containing 100 mg L^−1^ of Fe and 10 mg L^−1^ of Mn and Zn (TraceCERT®, Sigma-Aldrich, St. Louis, Missouri, USA) were used to prepare the calibration standards in 1% (vv^−1^) ultrapure nitric acid. Two 7-points external calibrations were performed using calibration solutions with concentrations of Cd, Fe, Mn, and Zn in the range 0.005–50 mgL^−1^ and 0.010–16 mg L^−1^ for Cd and Fe, respectively, and 0.001–1.6 mg L^−1^ for Mn and Zn.

The Zn, Cd, Mn, and Fe quantification was carried out choosing the wavelengths of 213.857, 214.439, 257.610, and 259.940 nm, respectively, as free of interferences.

### Alkaline comet assay

Plant cell nuclei for the alkaline comet assay were obtained from *A. thaliana* and *N. caerulescens* GA fresh leaves directly taken from plants in hydroponic culture. A 60-mm Petri dish was used to keep leaves on ice before the execution of the test. Leaves were cut with a razor blade, perpendicularly to the main rib. The cut surface was sprinkled with 200 µL of 0.7% low melting point agarose (LMA) in phosphate-buffered saline (PBS) to collect nuclei. Afterwards, nuclei were transferred to a precoated glass slide (1% normal melting point agarose in PBS), covered with a cover slip and cooled at 4 °C for minimum 15 min. A final layer was obtained using 90 µL of 0.7% LMA in PBS and cooling again at least 15 min at 4 °C. After the cover slips remotion, the slides were moved to an electrophoresis apparatus, and a cold electrophoresis buffer (1 mM Na_2_EDTA, 300 mM NaOH, pH ≥ 13) was added. A 15-min incubation in the electrophoresis buffer allowed the DNA unwinding prior to turn on electrophoresis (0.66 V cm^−1^, 300 mA, 15 min, 4 °C). After the electrophoresis, slides were treated with a neutralization buffer (0.4 M Tris–HCl, pH 7.5), fixed 5 min in cold 96% ethanol, and dried at RT. Nuclei, stained with 75 µL ethidium bromide (10 µg mL^−1^), were observed through a fluorescence microscope (Leica DMLS, Leica Microsystems, Wetzlar, Germany) (excitation filter: BP 515–560 nm; barrier filter: LP 580 nm), equipped with a monochromatic camera (Pulnix PE-2020P, Pulnix, Alzenau, Germany). An automatic image analysis system (Comet assay IV; Perceptive Instruments Ltd., Bury St. Edmunds, UK) was used to analyze fifty random selected nuclei per slide. Two slides were analyzed for every tested condition. Tail moment, that considers the migration of the genetic material as well as the relative amount of DNA in the tail, was chosen as parameter to evaluate nucleoid structure modifications.

### Methy-sens comet assay

An enzymatic restriction reaction was performed on the plant cell nuclei, fixed on glass slides (see paragraph “Alkaline comet assay”), using FastDigest *Msp*I and *Hpa*II enzymes (Thermo Fisher Scientific). Both endonucleases recognize CpG isles, but HpaII cut only the demethylated ones. The test was performed as described by Perotti et al. (2015), with minor modifications. Nuclei fixed on slides were dipped into PBS (10 min, RT), placed horizontally and sprinkled with 100 μL enzymatic solution, containing either *Msp*I or *Hpa*II diluted in FastDigest Buffer. Slides were covered with a coverslip and incubated for 10 min at 37 °C. FastDigest buffer (100 μL) was used as a negative control. Slides were, then, transferred into the electrophoretic chamber, covered with the electrophoretic buffer (1 mM Na_2_EDTA, 300 mM NaOH, pH ≥ 13). A 30-min electrophoresis (0.66 V cm^−1^, 300 mA) was performed and slides were neutralized, fixed, and stained as described above. Nuclei were observed through a fluorescence microscope and tail moment was chosen as reference parameter. The reaction with *Msp*I produce the maximum of the fragmentation observable and represents the positive control. The fragmentation induced through *Hpa*II enzyme is inversely proportional to the DNA methylation status.

### Superoxide dismutase (SOD) activity

SOD activity was evaluated by native polyacrylamide gel electrophoresis and in-gel NBT Staining. Native soluble proteins were extracted from leaves harvested from *N. caerulescens* GA and *Arabidopsis thaliana* plants, submitted to treatment with 50 μM or 5 μM CdSO_4_ (for *N. caerulescens* and *A. thaliana* respectively) for 1 week. Fresh tissues were grinded in 0.15 M Tris, pH 7.5. After centrifugation, the supernatant was recovered and the total protein content was estimated using the Bradford protein assay (Bio-Rad Laboratories, Hercules, CA, USA). Proteins (40 μg total protein) were separated by native 10% poly-acrylamide gel electrophoresis conducted at 4 °C in Tris–glycine native buffer (25 mM Tris, 0.192 M glycine). In-gel SOD activity was determined by incubating the gels in 1.23 mM NBT in the dark and afterwards in Temed-riboflavin (28–0.028 mM), as described in Chu et al. (2005). SOD activity is detected upon illumination with 40 µmol m^−2^ s^−1^ of white light as white spots in a purple background. Coomassie staining of replica gels demonstrated equal protein loading.

### Quantification of H_2_O_2_, 8-oxo-dG, and O_2_˙^–^ in intact leaves

Hydrogen peroxide present in the green tissues was determined following the protocol described by Junglee et al. 2014, with minor modifications: 50 mg of frozen tissues, previously harvested from *N. caerulescens* GA and *Arabidopsis thaliana* plants, submitted to treatment with 50 μM or 5 μM CdSO_4_ (for *N. caerulescens* and *A. thaliana* respectively) for 1 week, homogenized in 400 mL of solution 1:2:1 = Trichloroacetic acid (TCA) (0.1% (w:v)): KI (1 M): potassium phosphate buffer (10 mM, pH 7) at 4 °C for 10 min. The homogenate was centrifuged 10 min, 15,000 × *g* at 4 °C, to precipitate cell debris 200 μL of supernatant from each tube were placed in UV transparent microplate wells and incubated 20 min at RT. Samples were repeated in triplicate. A calibration curve obtained with H_2_O_2_ standard was used for quantification. The Infinite 200 PRO—Tecan For Life Sciences microplate reader was used to measure A_350_ nm.

8-oxo-dG was quantified on genomic DNA extracted from 50 mg of frozen tissues, previously harvested from *N. caerulescens* GA and *Arabidopsis thaliana* plants, submitted to treatment with 50 μM or 5 μM CdSO_4_ (for *N. caerulescens* and *A. thaliana* respectively) for 1 week. DNA was isolated by grinding the tissue in liquid nitrogen to fine powder. Upon liquid nitrogen evaporation, isolation buffer was added (0.3 M NaCl, 50 mM Tris–Cl pH7.5, 20 mM EDTA, 0.5% SDS). After 5-min incubation at RT, an equal volume of PCI (Phenol:Chloroform:Isoamyl Alcohol, 25:24:1) was added. After centrifugation, DNA was precipitated adding 0.8 V isopropanol to the upper phase and recovered by centrifugation at 12.000 g for 5 min at 4 °C. The DNA pellet was washed in 75% cold ethanol and after brief air-drying, resuspended in water. Four micrograms of DNA was digested with a nuclease mix that degrades DNA to its individual nucleoside components (DNA Degradase Plus, Zymo Research, CA, USA). Base quantification was done with UHPL-MS/MS (ExionLC-API 6500 + , Sciex). Chromatographic separation was performed in elution gradient (Phase A 10 mM HCOOH pH 3.75, Phase B MeOH) with column (ATLANTIS C18, Waters). The detection was performed in MRM mode and positive ionization.

In situ localization of O_2_˙^–^ was determined by treating leaves with NBT and visualizing the blue spots (Rao and Davis 1999): plants were grown in hydroponic culture and treated with CdSO_4_ as described above. Directly after harvesting, leaves were vacuum infiltrated with 10 mM potassium-phosphate buffer at pH 7.8. Immediately leaves were treated with 0.1% NBT in 10 mM potassium phosphate buffer, pH 7.8 for 20 min at room temperature. Stained leaves were cleared by boiling 80% ethanol and photographed. Blue precipitations correspond to sites where O_2_˙^–^ is accumulated.

### RNA isolation and qRT-PCR

Plant material was the same as for the experiments described above. Total RNAs were purified from frozen tissue using the TRIzol reagent (Thermo Fisher Scientific), according to the manufacturer’s instructions. 1.5 μg of total RNA was treated with DNAse and then submitted to first-strand cDNA synthesis following the Superscript II Reverse Transcriptase Kit (Thermo Fisher Scientific); oligo-dT was adopted as primers.

Real-time RT-PCR was performed using the Platinum SYBR Green qPCR SuperMix-UDG kit (Thermo Fisher Scientific) in the StepOnePlus Real-Time PCR instrument (Applied Biosystems). Each reaction (40 amplification cycles) was carried out in triplicate and a melting curve protocol was applied at the end of the runs to confirm amplification specificity. Relative expression for each gene was evaluated using the 2^−ΔΔCT^ method, and the expression level detected in the control sample was set as 1 (Livak and Schmittgen 2001). Primers for *MET1*, *LOX1*, *DDM1*, *DRM2*, and *RHD2* targets are listed in Supplementary Table S1. The target genes were selected within the *N. caerulescens* transcriptome shotgun assembly (TSA) based on the homology with *A. thaliana* gene sequences available in GenBank; if possible, the same primer pairs were designed for both species on conserved regions. For evaluation of *IRT1* expression, plant material of both shoots and root tissues was collected after 1-week treatment, as described above. *IRT1* specific primers were designed based on sequence information reported on the TAIR website for *AtIRT1* and in Halimaa et al. 2019 for *NcIRT1*. The gene specific primer pairs, designed using the online tool Primer3 (http://primer3.ut.ee), were purchased from BMR Genomics (Padua, Italy). Each primer pair was tested to assess its efficiency and specificity. Efficiency was determined with the standard curve method (data not shown), while specificity was verified by sequencing of the fragments obtained with the amplification of *N. caerulescens* GA and *A. thaliana* cDNAs. Endogenous reference genes for data normalization were Tubulin (AT1G04820) and Tubulin homologue (GFUL010929527) for *A. thaliana* and *N. caerulescens* GA respectively.

### Statistical analyses

Statistical analyses were performed using SPSS 21.0.0 software for Windows (© Copyright IBM Corporation 1989, 2012). Student’s *t* test was used to evaluate metal contents and comet assay results. Expression analysis results were analyzed by one-way ANOVA followed by a post hoc Tukey’s test.

## Results and discussion

### Metal content in leaf tissues

*N. caerulescens* GA was grown in hydroponic solution without Cd addition (control) or in Hoagland’s solution contaminated with 50 μM Cd, for 1 week. Cd accumulation was measured in shoots and control plants shows an average Cd content of 740 μg g^−1^ DW (Table 1). In Cd-treated plants, the content of Fe, Zn, and Mn in shoots was significantly lower than in untreated plants. Leaves of *A. thaliana* grown for 1 week in hydroponic solution contaminated with 5 μM Cd showed 215 μg g^−1^ DW Cd accumulation, corresponding to approximately three times lower Cd accumulation than in the leaves of the hyperaccumulator *N. caerulescens* GA. Also, a significant increase in Fe, Zn, and Mn in shoots is reported (Table 1). While no visible symptoms of chlorosis or toxicity were observed in *N. caerulescens* GA plants, *A. thaliana* plants treated with Cd showed initial symptoms of chlorosis in the leaves after 7 days of treatment and died after 4 weeks of treatment (Suppl. Figs. S1, S2).

**Table 1:** Metal content µg g^−1^ DW (mean ± SD) in leaves in *Arabidopsis thaliana and Noccaea caerulescens* (Ganges) treated with Cd

Species	Growth Condition	Sample	Cd	Fe	Zn	Mn
*A. thaliana*	- Cd	At	n.d.	142 ± 1	62.3 ± 0.5	123 ± 1
	5 μM Cd	At + Cd (5 days)	215 ± 1.3	306 ± 3.5**	101.3 ± 1.1**	158 ± 0.5**
*Noccaea caerulescens* (Ganges)	- Cd	Nc	n.d.	85.3 ± 0.9	138.6 ± 0.7	230.1 ± 3.5
	50 μM Cd	Nc + Cd (1 wk)	740 ± 5.3	37 ± 0.2**	91.18 ± 0.4***	196.33 ± 1.5**

Asterisks correspond to statistically different values respect to the control (Student’s t test, **p ≤ .01; ***p ≤ .001). Abbreviation: *n.d.*, not detectable

The reduction in leaf content of Fe, Zn, and Mn on metal hyperaccumulator species, upon Cd treatment, has been previously reported in similar experiments (Halimaa et al. 2019). This could be explained considering a competition between Cd and nutrient elements for the putative transporters. Among the membrane transporters involved in the translocation of metal ions, such as Mn, Fe, and Zn, IRT1 has been pointed to play a particularly important role (Connolly et al. 2002). Even if mainly specific for Fe, IRT1 could be responsible also for entrance of Cd (Halimaa et al. 2019). Indeed, in Cd exposed *N. caerulescens* GA plants, the expression of *IRT1* is enhanced by Cd in roots while it is barely observed in shoots (Suppl. Fig. S3). In *A. thaliana* plants a different situation is observed, being *IRT1* expressed at low level in roots, but induced in the shoot by Cd application. This overexpression might be the cause of the increased accumulation in the shoot of Fe, Zn, and Mn showed by the Cd-treated *A. thaliana*. This is in apparent contradiction with previously published results, in which *IRT1* seems not to be expressed in *A. thaliana* shoots upon Cd treatment (Connolly et al. 2002). It must be noted that the previous experiments have been conducted on *A. thaliana* seedlings, placed in Fe-deficiency medium, and treated with 90 µM for up to 72 h (Connolly et al. 2002). It is possible that such different conditions are not comparable to the experimental conditions applied in this work, mainly considering that we have treated *A. thaliana* plants with 5 µM Cd, which is already harmful (see toxicity symptoms after 4 weeks of treatment in Suppl. Fig. S2).

### DNA damage induced by Cd on leaf nuclei

**Fig. 1:**
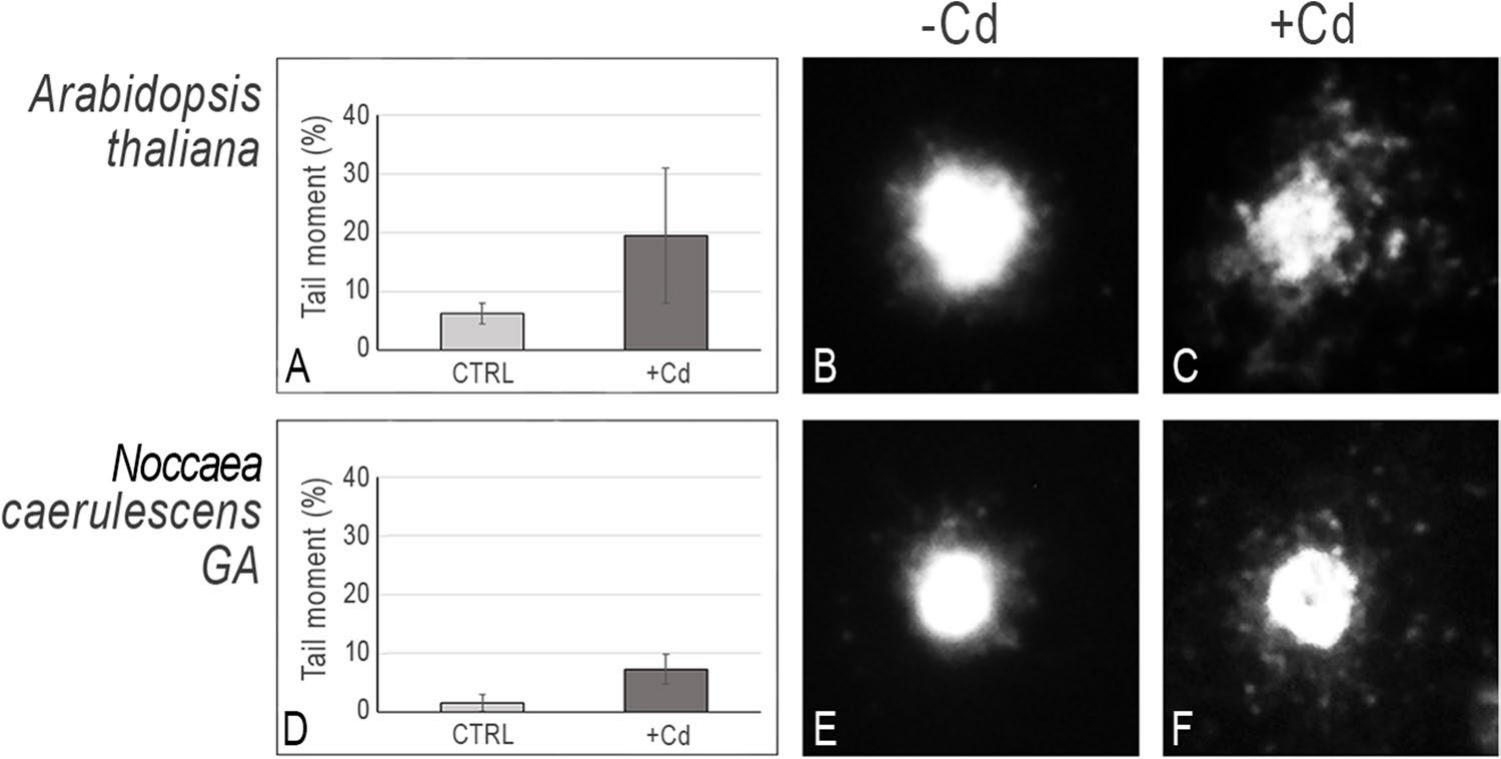
Alkaline comet assay on *A. thaliana* and *N. caerulescens* Ganges leaf nuclei. **(A, D) **Tail moment percentage (50 nuclei/duplicate slide); **(B, E**) Comet images of nucleoids extracted from leaves of untreated (CTRL) *A. thaliana* and *N. caerulescens* Ganges plants; (**C, F**) Comet images of nucleoids extracted from leaves of Cd treated (+ Cd) *A. thaliana* and *N. caerulescens* Ganges plants, as described in M&M; CTRL, plant grown in standard hydroponic solution; + Cd, plant grown in the presence of 5 μM for A. thaliana or 50 μM CdSO_4_ for *N. caerulescens* for one week. Images were captured at 400 ×

Treated and control *A. thaliana* and *N. caerulescens* GA plants were subjected to leaf nucleoids extraction, and DNA integrity was analyzed exploiting the alkaline comet assay. Figure 1 shows the different sensitivity to Cd between the non-tolerant and the hypertolerant species. Interestingly, a relevant enhancement of DNA migration was detected *in A. thaliana* after Cd treatment, in comparison with the untreated control (Fig. 1A). On the contrary, only a little increase in terms of tail moment was observed in Cd-treated *N. caerulescens* GA (Fig. 1D). The comet assay has been widely used to evaluate the effects of heavy metals in plants. It has been used, for instance, to evaluate the effects of Cd exposure in *Allium cepa* and in tomato, or Pb in pea (Seth et al. 2008; Pizzaia et al. 2019). This assay enables to detect DNA damage even at low concentrations of contaminants (Rodriguez et al. 2019) and allows the identification of modifications in the DNA that alters the supercoiling of the molecule, since loops move towards the anode during electrophoresis (Tice et al. 2000). In a previous work, the comet assay was employed to compare the effects of Cd exposure on the integrity of leaf cell nuclei in the Cd hypertolerant and hypercaccumulator species *Arabidopsis halleri* PL22 vs the non-tolerant species *A. thaliana,* showing a highly condensed nucleus in the hyperacculator species upon Cd treatment and a nucleus degradation in the non-tolerant one (Galati et al. 2021). A similar result was observed in the Ni hyperaccumulator *N. caerulescens* Monte Prinzera population vs the non-tolerant *A. thaliana*. We showed that upon Ni treatment, the nuclear structure was more compact in the hyperaccumulator species, if compared to the non-tolerant control species (Gullì et al. 2018). Overall, these results suggest the existence in hyperaccumulator species of a defence mechanism preventing genome instability and direct Cd-induced damage to the DNA structure.

### Variation of DNA methylation due to Cd exposure

**Fig. 2:**
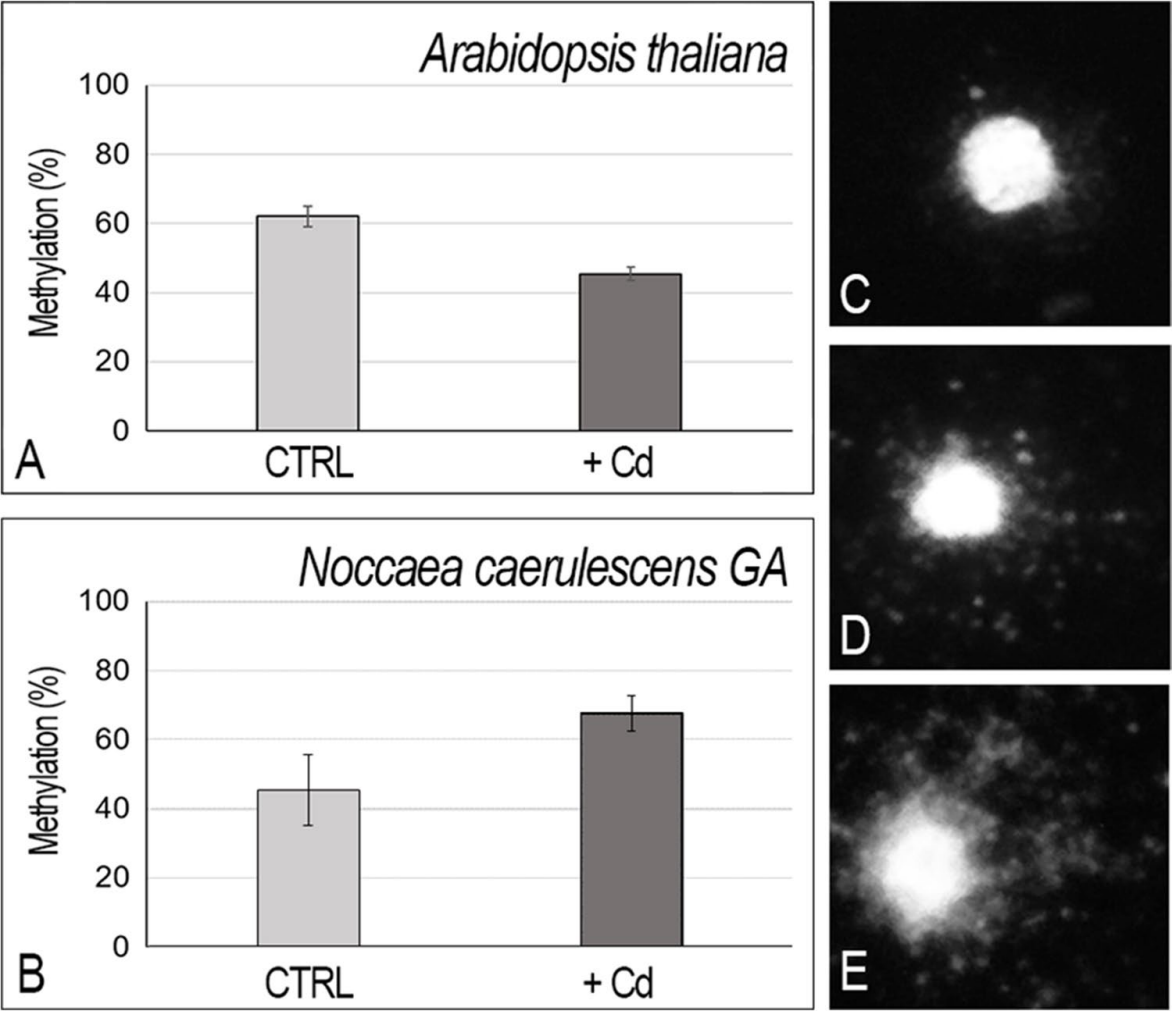
Methy-sens comet assay on nucleoids from leaves of *A. thaliana* and *N. caerulescens* GA untreated (CTRL) or treated with Cd as described in M&M (+ Cd). **A**,** B** Percentage of methylation after Cd treatment assessed through enzymatic digestions (100 − TI%[HpaII]/TI% [MspI] * 100)), in *A. thaliana* (**A**) or in *N. caerulescens* GA (**B**); (**C**,** D**,** E**) example of the undigested (**C**), digested with *Hpa* II (**D**), or *Msp* I (**E**) nucleoids of *N. caerulescens* GA. Images were captured at 400 ×

The methy-sens comet assay has been adopted as an informative technique to detect the global methylation modifications at level of single cell (Perotti et al. 2015). The method is based on methylation sensitive differential DNA enzyme digestion, i.e., *Msp*I (insensitive to methylated CpG sites) and *Hpa*II (sensitive to methylated CpG sites). The methy-sens comet assay was performed on nuclei extracted from leaves of *A. thaliana* and *N. caerulescens* GA plants treated with Cd to explore the modulation of CpG methylation in response to the presence of this heavy metal. In the absence of Cd, *A. thaliana* presented a higher basal level of methylation (62%) than the metal-tolerant species *N. caerulescens* GA (45%) (Fig. 2A, B). A decrease in the methylation percentage, about 17%, was observed in *A. thaliana* (Fig. 2A) upon Cd treatment. On the contrary, a significant increase in methylation, about 23%, was detected in *N. caerulescens* GA (Fig. 2B). This data may be related to a less damage of the DNA molecule in the hypertolerant and hyperaccumulator species vs the non-tolerant and non-accumulator plant species as shown in Fig. 1. An important increase in methylation was detected in a previous work in *A. halleri* PL22 population upon Cd exposure (Galati et al. 2021). These data support the hypothesis that Cd treatment can induce DNA modifications in hyperaccumulators, which include the amount of methylated DNA as epigenetic modifications. Interestingly, such methylation change is highly dependent on the species considered. Indeed, a general increase in methylation has been also reported in pokeweed, after Cd addition (Jing et al. 2022). Conversely, a similar treatment did not show any modulation in methylation level in soybean (Holubek et al.^ 2020^), pointing to the fact that generalization must be made with care, and different genotypes behave differently when challenged with heavy metals. Also, the rice methylome has been investigated in response to Cd exposure, highlighting that Cd tended to reduce the overall DNA methylation levels also in this Cd-sensitive species (Feng et al. 2016).

### Antioxidant activity and ROS production

**Fig. 3:**
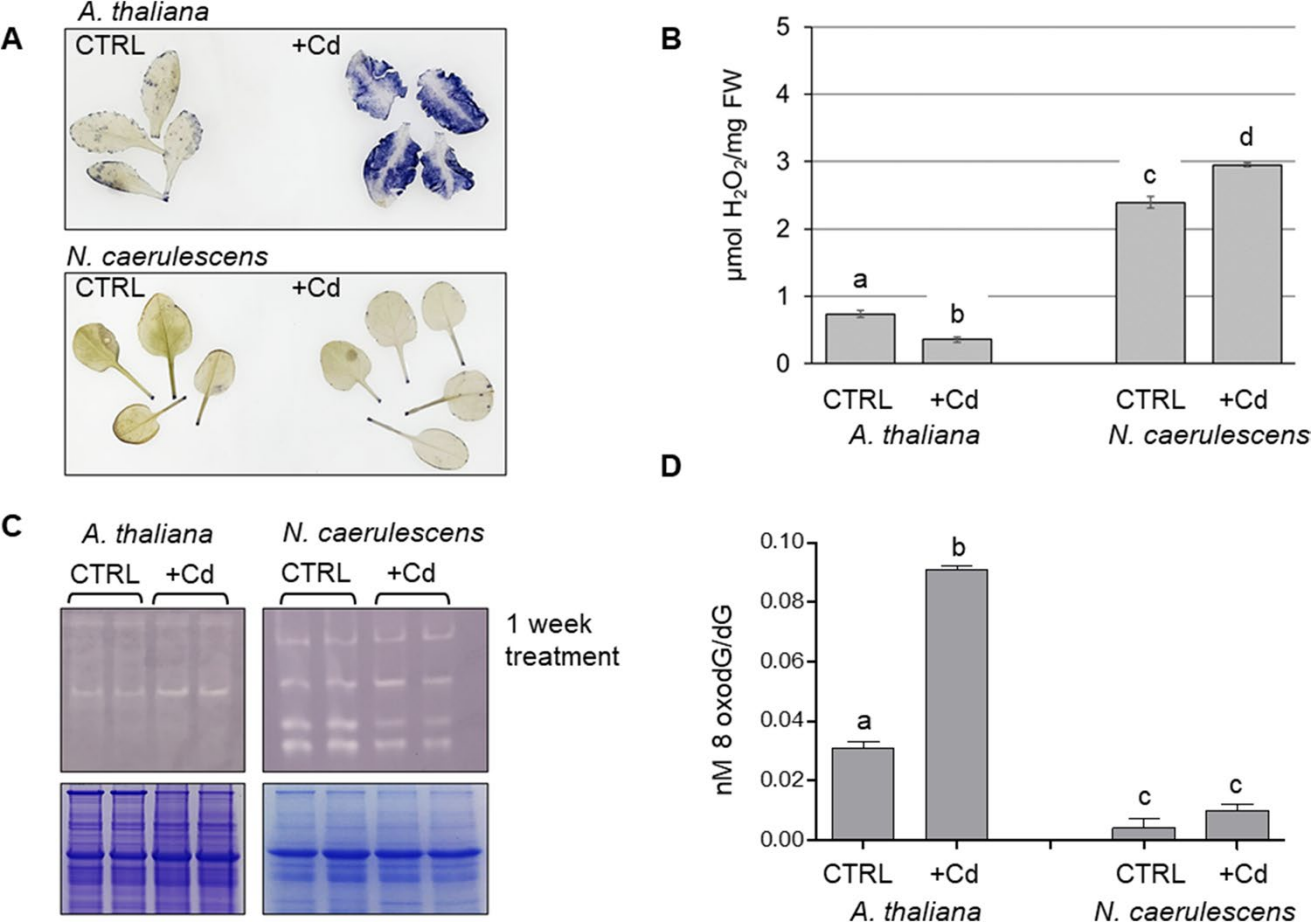
**A** NBT staining to detect O_2_˙.^–^ in the intact leaves of 5-wk-old plants grown under standard conditions (CTRL) and in hydroponic solution supplemented with 50 and 5 µM Cd (+ Cd) (for *N. caerulescens* and *A. thaliana* respectively) for 2 weeks. In both experiments, leaves were harvested in the morning, before the light of the growth chamber was switched on. **B** H_2_O_2_ quantification in leaves of *N. caerulescens* Ganges and *A. thaliana* plants kept in hydroponic conditions under standard solution (CTRL) or treated for 1 week with 50 µM Cd and 5 µM Cd (+ Cd) respectively. H_2_O_2_ concentration, expressed as nmol/mg FW was evaluated by using a calibration curve obtained with H_2_O_2_ standard solutions prepared in 0.1% TCA. Each value represents mean ± standard deviation, and the same letter corresponds to non-statistically significant differences evaluated by one-way ANOVA followed by a post hoc Tukey's test. **C** Quantification of SOD activity, on 40 μg of total proteins: upper panel, in-gel analysis of SOD activity on *N. caerulescens* Ganges and *A. thaliana* plants kept in hydroponic conditions under standard solution (CTRL) or treated for one week with 50 µM Cd and 5 µM Cd (+ Cd) respectively; lower panel, Coomassie staining of the replica gels in SDS-PAGE showing comparable loading between lanes. For each condition, both control and + Cd, two biological replicates were included in the analysis and are reported in the figure. **D** Quantification of 7,8-dihydro-8-oxo-2′-deoxyguanosine (8-oxodG) on 4 µg of DNA digested to its individual nucleosides as described in M&M. Each value represents mean ± standard deviation, and the same letter corresponds to non-statistically significant differences at *P* < 0.001

Cd is usually highly toxic to non-tolerant plants. *A. thaliana* is greatly affected by Cd addition to the culture medium and stops growth and development, and shows an extreme chlorotic phenotype and does not survive after 4 weeks (Suppl Fig. S2). Five days of 5 µM Cd treatment was enough to greatly enhance ROS production in leaf tissues, as determined by in situ localization and relative quantification of O_2_˙^–^ (Fig. 3A). Such elevated generation of superoxide anion in leaves of Cd-treated *A. thaliana* could be a direct effect of Cd toxicity on plant cells. Interestingly, Cd treatment of *N. caerulescens* GA does not reflect an increase of reactive oxygen moiety O_2_˙^–^, as showed in Fig. 3A and plants do not show signs of Cd stress (Suppl. Figs. S1, S2). Considering also other molecules that have been commonly identified as oxidative stress biomarkers, the amount of H_2_O_2_ has been evaluated in control and Cd treated plants. Interestingly, even though Cd induces a high amount of superoxide anion, measured by NBT staining, the amount of hydrogen peroxide was fairly lowered by Cd treatment in *Arabidopsis* leaves (Fig. 3B). In the hyperaccumulating species the opposite happens, and H_2_O_2_ accumulated in response to Cd (Fig. 3B). ROS generation is counteracted by the antioxidative system, made up by SOD, and other enzymes responsible for ROS scavenging. In our SOD assay, Cu/Zn-SOD activity in *A. thaliana* was unaltered by Cd treatment (Fig. 3C) (comparing the lane intensity also with the Coomassie staining of the gel). This could be interpreted as an interference of Cd itself with the antioxidant enzymes or it could be ascribed to a Cd-induced Zn deficiency in tissues (Drazkiewicz et al. 2007), or because of the Cd-induced downregulation of the Cu/Zn SOD expression, as observed in other species (Li et al. 2017). Similar experiments on leaf tissue of *A. thaliana* treated with Cd for 7 days reported a decrease in Cu/Zn-SOD activity (probably ascribed to a Cd-induced Zn deficiency in tissues), while Mn- and Fe-SODs are greatly enhanced (Drazkiewicz et al. 2007). Such Cd effect on SOD activity has been demonstrated also in other sensitive species, such as *Phaseolus vulgaris* or *Pisum sativum*, attributing to Cd both the effect of reduction in the activity of antioxidative enzymes and the varying accumulation of different ROS species (Romero-Puertas et al. 2007). Regarding the activity of superoxide dismutase, in *N. caerulescens* GA, Cd does not lead an enhanced SOD activity, as showed by the faint bands in the gel of Fig. 3C. Similar behavior of SOD activity was also detected upon treatment of other hypertolerant and hyperaccumulator species, such as *Phytolacca americana* in which Cu/Zn-SOD expression is reduced by Cd application (Jing et al. 2022). The contribution of other scavenging enzymes must be taken into account, for instance catalases (CAT), peroxiredoxins (Prxs), and glutathione peroxidase (GPX), whose activity is to conversion of H_2_O_2_ to water, and depends on the availability of non-enzymatic compounds (e.g., ascorbate and glutathione). Mass spectrometry analysis of the lower bands collected from the SOD-activity gel has shown that within this gel position a variety of glutaredoxin-dependent peroxiredoxin and thioredoxin-dependent peroxiredoxins are present (data not shown). Interestingly, the activity of this ROS-scavenging system is reduced in Cd-treated *N. caerulescens* GA plants, and this could contribute to the enhanced hydrogen peroxide accumulation in this species upon Cd treatment (Fig. 3B). Due to its remarked stability in the plant cell, hydrogen peroxide is considered the main ROS with signaling activity (Huang et al. 2019). Indeed, H_2_O_2_ is thought to mediate a tight connection between the redox status and the epigenetic processes, being involved in modulating DNA methylation (Shen et al. 2016). In pokeweed, the level of H_2_O_2_ has been correlated to the activity of methylase and demethylase genes, contributing to modify the methylated loci. The authors concluded that the metal-induced ROS may inhibit DNA methylation. Interestingly, rather than the overalls methylation levels, it is the site-specific hypomethylation patterns, the key response of *Phytolacca americana* to Cd stress (Jing et al. 2022). Also, in the aquatic macrophyte *Hidrophylla verticillata*, DNA de-methylation was induced by excess Cu, and such event could be reversed by the pretreatments with NADPH oxidase inhibitors, again suggesting a link between de-methylation changes triggered by excess Cu and ROS production (Shi et al. 2017). Therefore, we would suggest that the difference in hydrogen peroxide accumulation between the hyperaccumulating species and the non-accumulating control maybe be differently involved in regulating the DNA methylation of the genome.

The guanine oxidation products, 8-oxoguanine (8-oxoG) and its nucleotide 7,8-dihydro-8-oxo-2′-deoxyguanosine (8-oxodG, also known as 8-OHdG) are the most common forms of cellular oxidative DNA damage, and notable biomarkers used to quantify the oxidative DNA damage in cells (Chiorcea-Paquim 2022; Valavanidis et al. 2009). We therefore measured 8-oxodG on DNA extracted to infer the oxidative damage due to ROS production in the plant cell. Interestingly, the 8-oxo-dG/dG ratio was significantly higher in the exposed *A. thaliana* plant than in the control, at *p* < 0.001, (Fig. 3D). The same ratio is also higher in *N. caerulescens* treated, but without reaching statistical significance. This would point to an increased DNA damage induced by Cd in the sensitive *A. thaliana*. Cd and Mn treatment of *P. americana*, induced 8-oxodG presence, which was abolished by pre-treatment with NADPH oxidase inhibitors (Jing et al. 2022). Interestingly, the ROS induced 8-oxo-dG has been proposed to interfere with the ability of DNA methyltransferases to bind the DNA, resulting in hypomethylations (Shi et al. 2017). We are still not able to say if the reduced methylation observed upon Cd treatment in *A. thaliana* is due to the increased amount of 8-oxo-dG, or it is a consequence of differential regulation of methylation-related genes induced by ROS production upon Cd stress. The mechanisms would differ in *N. caerulescens* GA, in which an increase in H_2_O_2_ content is accompanied by an enhanced DNA methylation and a reduction in DNA damage.

### Gene expression analysis

**Fig. 4:**
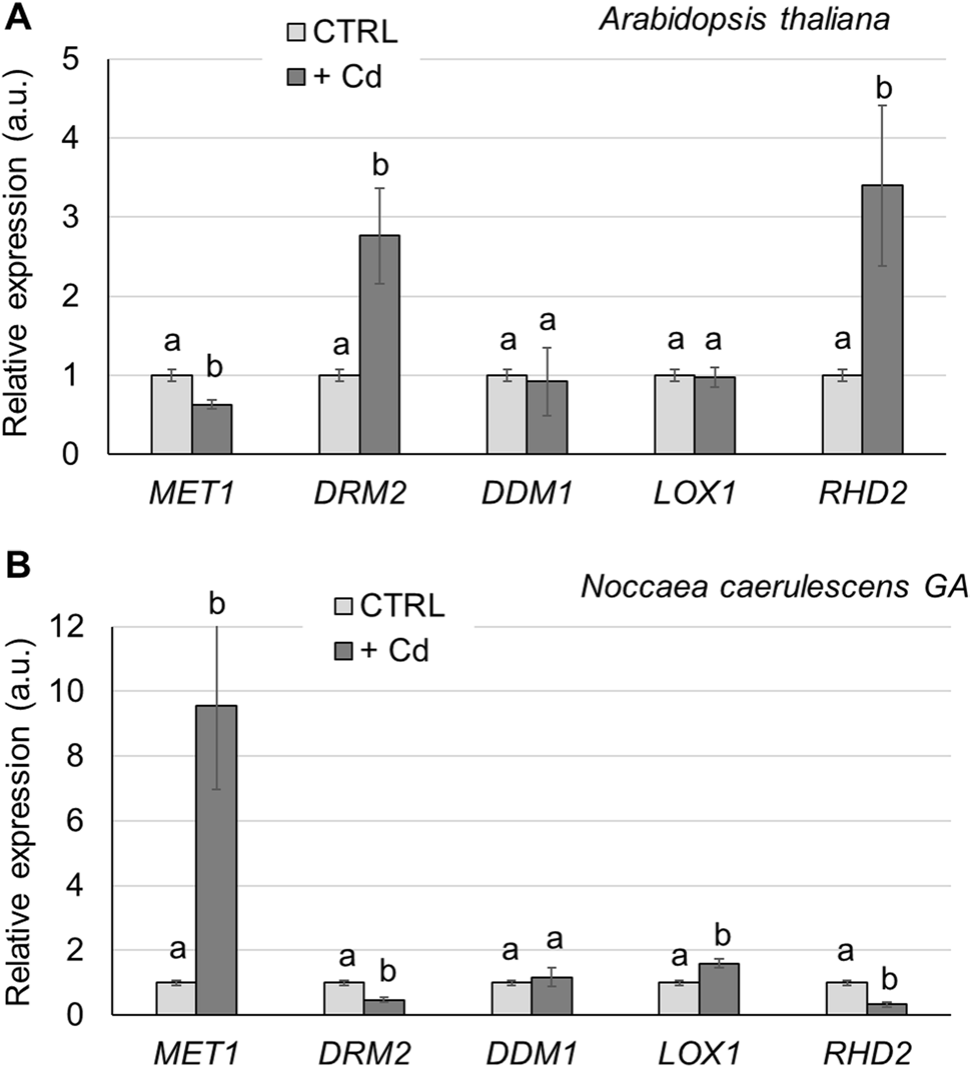
Real-time RT-PCR analysis on the expression of epigenetics-related genes *MET1, DRM2 DDM1,* and oxidative stress genes *LOX1, and RHD2.* **A** Relative expression in leaves of *A. thaliana* plants, treated for 1 week with 5 µM Cd in hydroponic solution. **B** Relative expression in leaves of *N. caerulescens* GA plants, treated for 1 week with 50 µM Cd in hydroponic solution. The same letter corresponding to non-statistically significant differences, evaluated within each gene samples, by one-way ANOVA followed by a post hoc Tukey’s test

The expression of genes involved in DNA methylation was evaluated in all samples of *N. caerulescens* GA and *A. thaliana* grown in control condition and in the presence of Cd (Fig. 4). In particular, the target genes were *MET1*, involved in methylation at CG dinucleotides (Kankel et al. 2003). The CG site is symmetrical on the opposite strand and MET1 maintains a pattern of methylation during DNA replication by a semiconservative mechanism; *DRM2*, coding for a member of Domains Rearranged Methyltransferases (DRM) involved in methylation in the non-symmetric CHH context (Cao and Jacobsen 2002) and *DDM*1 a gene coding for a chromatin remodeling ATPase which is required for correct DNA methylation (Jeddeloh et al. 1999). Two genes involved in antioxidant response were also analyzed. *LOX1* coding for a Linoleate 9S-lipoxygenase 1 and *RHD2* coding for respiratory burst oxidase homolog protein C both involved in Cd stress signalling from roots to shoots as well as signalling processes within the leaves (Keunen et al. 2013; Skórzyńska-Polit et al. 2006).

In *A. thaliana*, the *MET1* expression decreased upon Cd treatment (*P* < 0.01) while *DRM2* transcript increased more than two-fold in Cd treated plants (*P* < 0.01); in *N. caerulescens* GA plants treated with Cd, *MET1* transcript increased more than 8 times respect to the untreated samples (Fig. 4) (*P* < 0.01) and *DRM2* decreased significantly in the same condition (*P* < 0.05). No significant variation in *DDM1* gene expression was observed in both species.

Changes in DNA methylation patterns both involving the entire genome and at specific loci of DNA are a well-known mechanism enabling higher plants to rapidly adapt in response to stress. On the other hand, the stress can lead to genome instability and the methylation event can protect against this (Akhter et al. 2021). Indeed, global DNA hypomethylation has been associated to genomic instability, leading to chromosomal aberrations, genome rearrangement, chromosome, and point mutations (Chen et al. 1998).

The precise role played by Cd in DNA methylation has not been fully revealed. Nonetheless, recent experimental results point to the evidence that changes in methyl-transferase enzyme and mis-regulated modulation of the genes known to be responsible for faithful maintenance of methylation patterns are the major causes of altered DNA methylation in plants (Aina et al. 2004; Ou et al. 2012). In *A. thaliana*, Cd induced genomic instability with an increase of genomic methylation both at CpG and CHG sites (Wang et al. 2016). In addition, Arabidopsis mutants, in which DNA demethylation is inhibited, showed an increase in global DNA methylation and an improved Cd tolerance coupled with improved iron nutrition (Fan et al. 2020). These data are in accordance with the work here presented: *A. thaliana* as a Cd non-tolerant species, induces de novo casual methylation modifications to respond to Cd inducing DNA instability.

On the contrary, the Cd hyperaccumulator *N. caerulescens* GA as other hyperaccumulators previously tested (Galati et al. 2021) showed an increase in global CpG DNA methylation, as a potential defense mechanism to preserve cells from the high Cd content in plant tissues, which is related also to the less damage observed by alkaline comet assay.

The results on the expression of genes involved in oxidative response (Fig. 4) confirmed the difference in the SOD activity response, the ROS accumulation H_2_O_2_ and 8-oxodG observed in the two species (Fig. 3). *RHD2* transcript showed a fourfold increase in Cd treated *A. thaliana* plants, while in *N. caerulescens* a down-regulation of the gene expression was observed upon Cd treatment. *LOX1* gene expression seems to be not modulated in *A. thaliana* in response to Cd while an almost double expression of the gene was observed in *N. caerulescens* GA subjected to Cd treatment respect to the control. In Arabidopsis, LOX1 and RBOHD are both involved in stress signaling from roots to shoots upon Cd treatment. It has been showed that leaves of Cd-exposed *A. thaliana* plants had a significantly higher LOX activity as compared to control plants (Skórzyńska-Polit et al. 2006). It could be possible that other *LOX* genes are up regulated in Cd treatment in the conditions here tested since Arabidopsis *LOX* gene family comprise 6 members (Bannenberg et al. 2009). Respiratory burst oxidase homolog protein C is known to be involved in ROS production both in roots and in leaves which mediate the Ca signaling in defense to Cd treatment in *Arabidopsis* (Gupta et al. 2017).

In *N. caerulescens* GA, the reduction of the expression of *RHD2* is in accordance with the absence of ROS production in leaves of the hyperaccumulator species while the increase in the expression of *LOX-1* transcript could be a defense mechanism rather than associated to ROS production. LOX activity can lead to not only increased lipid peroxidation, but also to the production of oxylipin bioactive compounds involved in growth, development, and responses to (a)biotic stress conditions (Howe and Schilmiller 2002; López et al. 2011).

## Conclusion

In this work, the alkaline comet assay was used as a biomarker for diagnosis of Cd stress in the *A. thaliana* non-tolerant vs the hypertolerant and hyperaccumulator *N. caerulescens* GA plants. The methy-sens comet assay was also employed to evidence possible variation in global methylation patterns in the two species upon Cd treatment. Our data suggest a possible role of epigenetic modifications in the Cd hypertolerant and hyperaccumulator *N. caerulescens* GA population to face high Cd shoot concentrations while preserving genome integrity.

The differences between *N. caerulescens* GA and *A. thaliana* regarding the DNA modification and the expression of genes coding for enzymes involved in DNA methylation support the hypothesis of different mechanisms to prevent the Cd-induced DNA damage which evolved in the hypertolerant and hyperaccumulator species. The link between ROS production and signature, probably different between hyperaccumulators and sensitive species, the variation in DNA damage, and the variation in DNA methylation at different sequence context has still to receive the proper attention in hyperaccumulating species. The same is true for the processes that are associated with methylation-dependent transcriptional regulation, and their functional consequences, which undoubtedly need further studies.

## Supplementary Information

Below is the link to the electronic supplementary material.

Supplementary file1 (DOCX 8481 KB)

Supplementary file2 (DOCX 16 KB)

## Data Availability

Data sharing is not applicable to this article as no datasets were generated or analyzed during the current study.
