# Supplementary material for: DNA methylation is enhanced during Cd hyperaccumulation in Noccaea caerulescens ecotype Ganges
Source: Environ Sci Pollut Res Int. 2022 Nov 10;30(10):26178–90. doi: 10.1007/s11356-022-23983-w (PMC9995422; doi:10.1007/s11356-022-23983-w)

**Suppl Figure 1:** *.Noccaea caerulescens* and *A. thaliana* plants after 1 weeks at 50 and 5 micromol Cd treatment respectively, and relative controls.


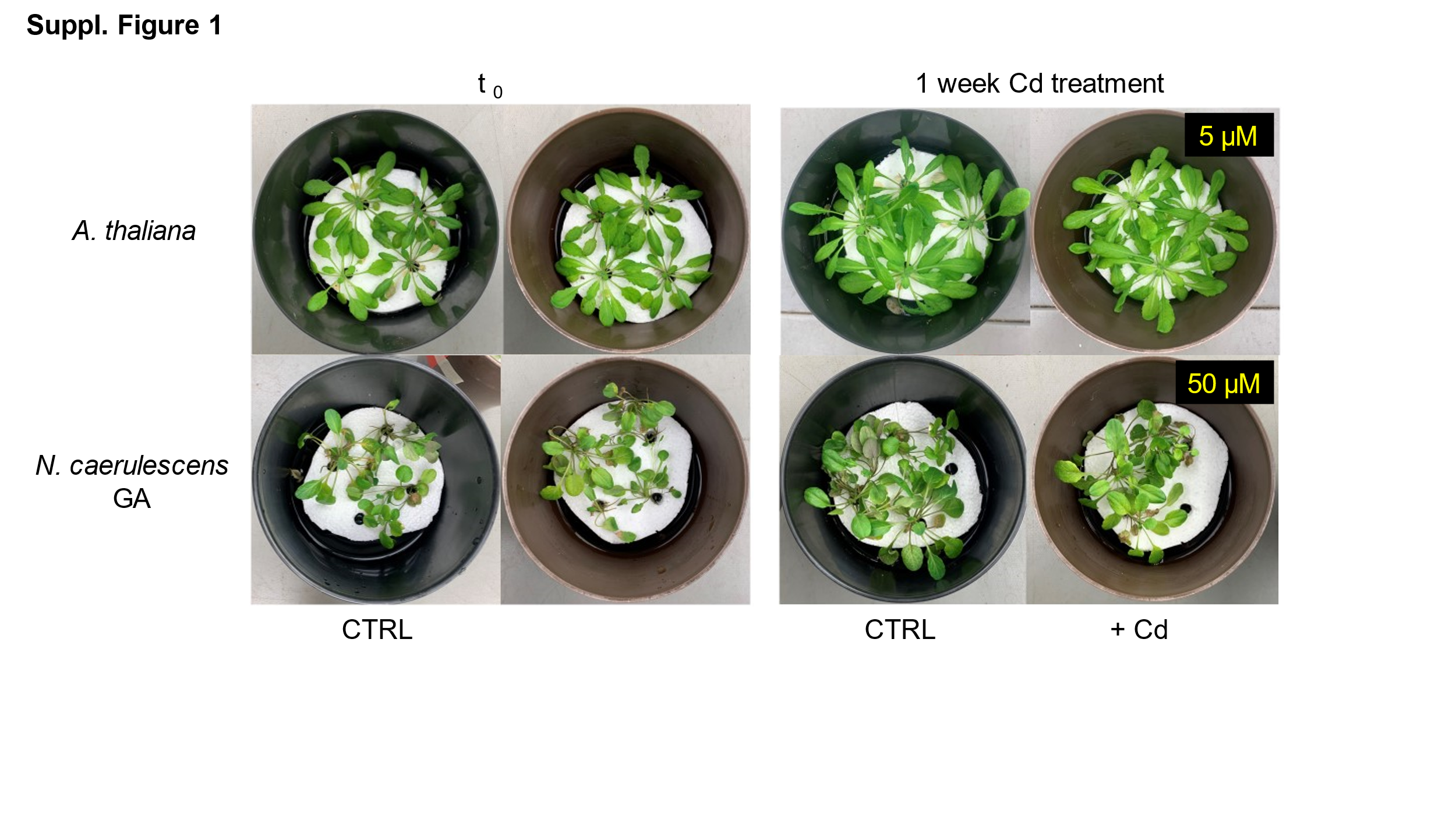


**Suppl Figure 2:** *.Noccaea caerulescens* and *A. thaliana* plants after 4 weeks at 50 and 5 micromol Cd treatment respectively, and relative controls.


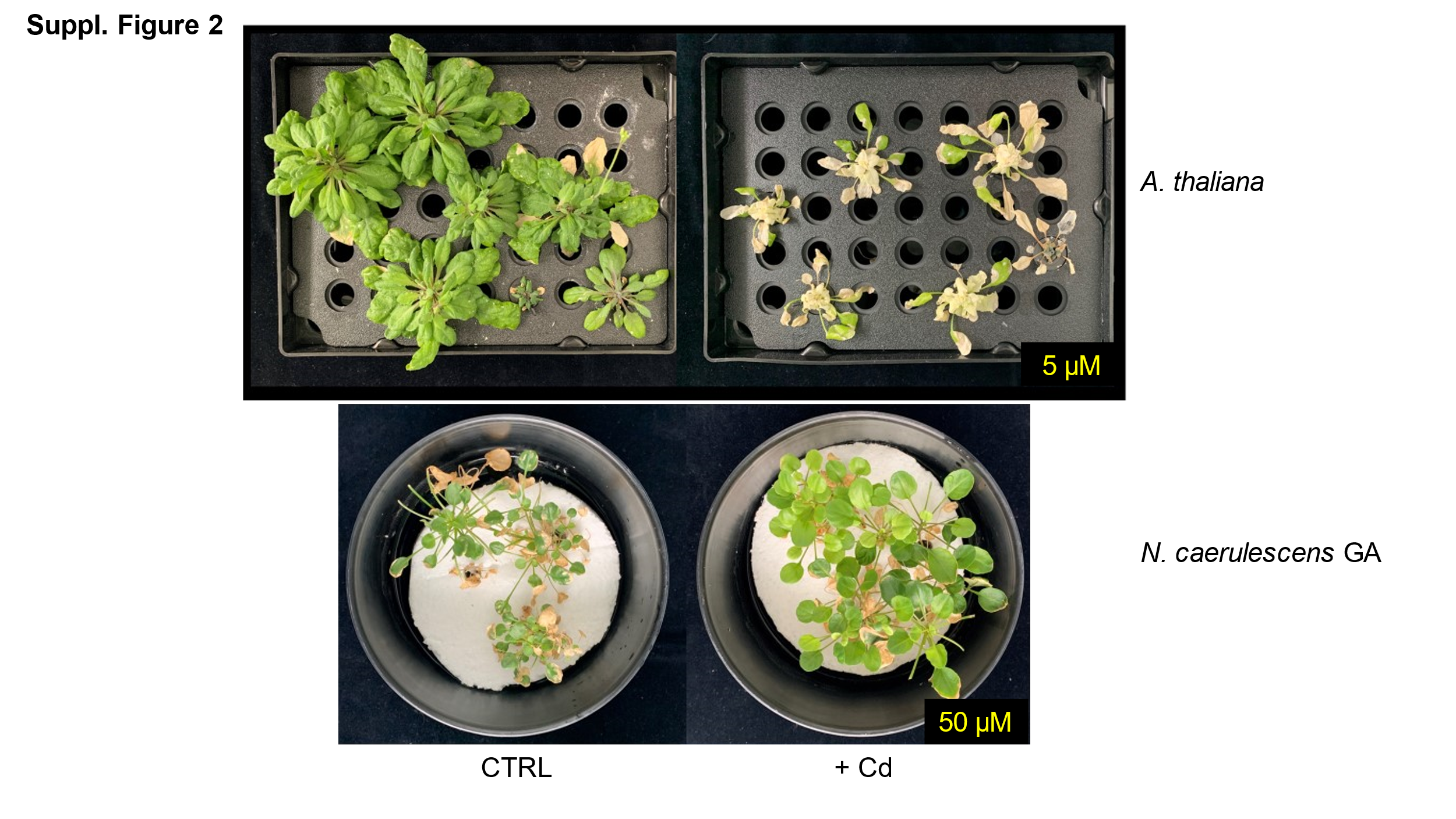


**Suppl Figure 3**: qRT-PCR analysis on the expression of IRT1 in shoots and roots of *Noccaea caerulescens* and *A. thaliana* control and Cd-treated plants. The samples were harvested after one-week treatment. The expression levels were calculated using the 2^−ΔΔCT^ method, expression level in roots of un-treated *Noccaea caerulescens* plants was set as 1. Statistically significant variations (P < 0.05) were evaluated by one-way ANOVA followed by a post hoc Tukey's test and are marked with letters, the same letter corresponding to non-statistically significant differences, evaluated within each gene samples.


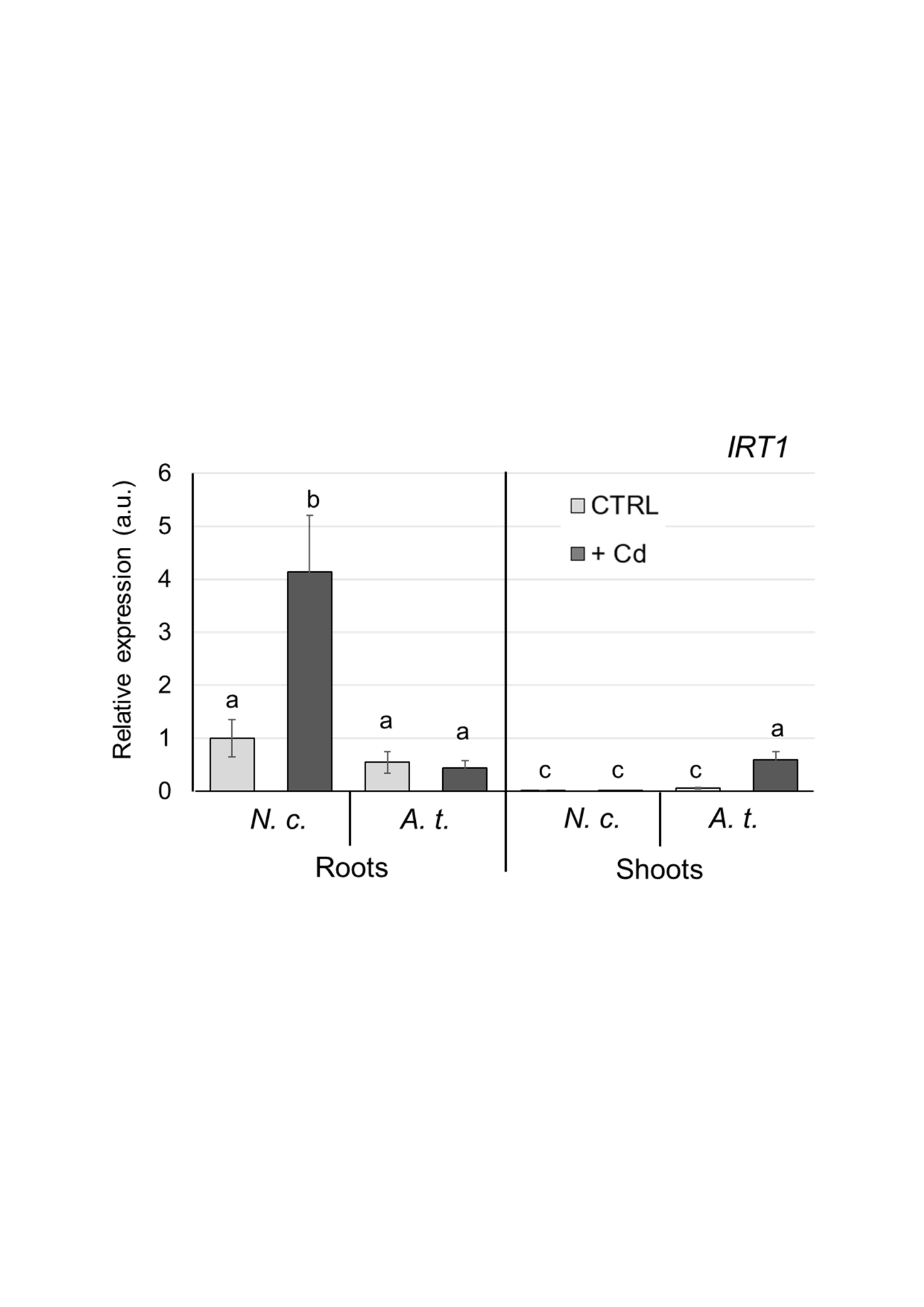

Supplement: Supplementary file 1 — Supplementary file1 (DOCX 8481 KB) [file 11356_2022_23983_MOESM1_ESM.docx]
