# Supplementary material for: DNA methylation is enhanced during Cd hyperaccumulation in Noccaea caerulescens ecotype Ganges
Source: Environ Sci Pollut Res Int. 2022 Nov 10;30(10):26178–90. doi: 10.1007/s11356-022-23983-w (PMC9995422; doi:10.1007/s11356-022-23983-w)
Supplement: Supplementary file 2 — Supplementary file2 (DOCX 16 KB) [file 11356_2022_23983_MOESM2_ESM.docx]

**Supplementary Table 1:** Genes analysed in this study and primers utilized for RT-qPCR

| **Gene name** | **Accession number (Ganges)** | **Function** | **Site affected** | **Primer sequences 5’-3’** | **Amplicon length (bp)** | |
| --- | --- | --- | --- | --- | --- | --- |
| *MET1 (N. c.)* | GEVM01028405.1 | DNA (cytosine-5)-methyltransferase 1 | CG | F- AAGGGAAGGGTGTGGAGAGT  R- GTTGCAGACACACCTGCTTG | | 145 |
| *MET1 (A. t.)* | [NM_124293.4](https://www.ncbi.nlm.nih.gov/nucleotide/NM_124293.4?report=genbank&log$=nucltop&blast_rank=2&RID=8S0ZFJKC013) | DNA (cytosine-5)-methyltransferase 1 | CG | F-AGCCTCTCTATCGACAAGCC  R-CCACAAAATAGATGGCCGGG | | 181 |
| *DRM2* | GEVM01014859.1 | DNA (cytosine-5) methyltransferase DRM2 | CHH | F- GCATCTGACGAGTTTCACGC  R- CGAACCTGCTCAACAACAGC | | 116 |
| *DDM1* | GEVI01022697 | ATP-dependent DNA helicase DDM1 | CG, CHG, CHH | F-ATGAGATTGCTAGGTTCACG  R-GCCGCAGACTTCTTTTAGCA | | 171 |
| *LOX1* | GEVI01028158 | Linoleate 9S-lipoxygenase 1 |  | F-TGGACTTTCCCTGACCAAGC  R-ACCTCAAGCCCATCCACTGC | | 131 |
| *RHD2*  *(RBOHC)* | GEVI01005584 | Respiratory burst oxidase homolog protein C |  | F-ACAGTGAAGCTGAACATGGC  R-ACTATAATCCCCACCGCTAT | | 146 |
| *TUB (N. c.)* | GFUL010929527 | tubulin alpha-4 chain |  | F-TAAAGACGTGAACGCAGCTGTT  R-TGAATCCAGTAGGACACCAGT | | 80 |
| *TUB (A. t.)* | NM_100360.4 | Tubulin alfa-4 chain |  | F-TGATGTACCGTGGTGATGTTGTC  R-CAAACTGAATAGTGCGCTTGGT | | 81 |

The following information is given for each primer pair: target gene name, accession number of *Arabidopsis thaliana* and *Noccaea caerulescens*, protein function, primer sequence, amplicon length (bp)
